# The Role of Social Stress in the Development of Inhibitory Control Deficit: A Systematic Review in Preclinical Models

**DOI:** 10.3390/ijerph18094953

**Published:** 2021-05-06

**Authors:** Lucía Sánchez-Salvador, Ángeles Prados-Pardo, Elena Martín-González, Manuela Olmedo-Córdoba, Santiago Mora, Margarita Moreno

**Affiliations:** Department of Psychology and Health Research Center (CEINSA), Almeria University-ceiA3, 04120 Almeria, Spain; sanchezsalvadorlucia@gmail.com (L.S.-S.); app717@ual.es (Á.P.-P.); emg771@ual.es (E.M.-G.); manoli.ocp@gmail.com (M.O.-C.); smp993@ual.es (S.M.)

**Keywords:** impulsivity, compulsivity, inhibitory control, social dominance, social stress, social isolation, addiction, aggressiveness

## Abstract

Inhibitory control deficit and impulsivity and compulsivity behaviours are present in different psychopathological disorders such as addiction, obsessive-compulsive disorders and schizophrenia, among others. Social relationships in humans and animals are governed by social organization rules, which modulate inhibitory control and coping strategies against stress. Social stress is associated with compulsive alcohol and drug use, pointing towards a determining factor in an increased vulnerability to inhibitory control deficit. The goal of the present review is to assess the implication of social stress and dominance on the vulnerability to develop impulsive and/or compulsive spectrum disorders, with the aid of the information provided by animal models. A systematic search strategy was carried out on the PubMed and Web of Science databases, and the most relevant information was structured in the text and tables. A total of 34 studies were recruited in the qualitative synthesis. The results show the role of social stress and dominance in increased drug and alcohol use, aggressive and impulsive behaviour. Moreover, the revised studies support the role of Dopaminergic (DA) activity and the alterations in the dopaminergic D1/D2 receptors as key factors in the development of inhibitory control deficit by social stress.

## 1. Introduction

Inhibitory control deficit is associated with impulsive and compulsive behaviours. Impulsive behaviour is defined as the tendency to act prematurely without foresight, often associated with addiction to substance use, attention-deficit/hyperactivity disorder (ADHD), mania and antisocial behaviour [1,2]. Moreover, compulsivity is defined as actions inappropriate to the situation that persist and have no obvious relationship to the overall goal, which often result in undesirable consequences [1,3,4]. It is present in obsessive-compulsive disorder (OCD) but also in addiction by compulsive drug-seeking and relapse [3,5]. Numerous studies have linked inhibitory control problems with socialization disorders [6,7,8]. It should be noted that people who suffer from OCD have more problems in their social and family relations, as well as greater difficulty finding and keeping a job [9,10]. Interpersonal relationships of people with OCD are marked by interpersonal ambivalence since they exhibit both prosocial and antisocial behaviour to the same extent [11,12]. However, few studies have assessed the role of social stress on the different facets of inhibitory control deficit.

In humans, social relations are governed by rules of social organization, which, in turn, affect our inhibitory behaviour. It has been found, for example, that one of the reasons adolescents play drinking games is to show their dominance over others [13]. In Asian university students, social dominance influences higher alcohol consumption [14]. In adults, it has also been found that those who consider it important to show their social dominance drink more [15]. Social and hierarchical distinctions seem to lead to the development of different coping strategies for stressors [16]. Stress can be defined in the field of experimental research as the exposure of the subject to an aversive stimulus or experimental situation [17]. Therefore, different ways of experiencing and dealing with stressors produce differences in the vulnerability of subjects to develop inhibitory control disorders. Exposure to stressful situations is associated with an increased likelihood of alcohol relapse in humans [18], as well as higher consumption of tobacco, alcohol and marijuana [19]. Additionally, experiencing vital stressful events during adolescence is related to alcohol consumption during adolescence and drug and alcohol consumption during adulthood [20]. Moreover, social stressors, such as problems at school or separation from parents, lead to higher drug use [21].

In animals, socialization is characterized by the relationship between individuals under patterns of hierarchy and submission. The dominant hierarchy of a group is defined as a fairly persistent, unequal ranking of members in terms of power, influence and access to valued prerogatives [22]. Once established, the hierarchy rank is relatively stable and can minimize intense fights among the group members [23]. In preclinical models, social stress can be assessed by different tests. One of the most relevant for studying social defeat is the Resident-intruder test (RIT), considered a stress induction protocol whereby stimulus rats, known as “residents,” show aggressive behaviour towards another unknown male rat called “intruder.” The procedure is based on introducing the “intruder” rat into the box of “resident” rats for 60 min. During this procedure, the resident female rat is removed from the cage. The “intruder” rat is housed in a wire mesh cage to ensure its protection but allows visual, auditory and olfactory contact. The “intruder” rat usually shows upright and crouching defensive positions, in addition to tracking the “resident” rat [24]. Another interesting model of social dominance is the Resource competition task (RCT); the rats designated as dominant using the RCT won more encounters than the subordinate ones. There are several social stress paradigms for assessing sociability in rodents. The most relevant is known as social isolation, which consists of housing rodents individually during the developmental period from weaning to adulthood. There are other paradigms, such as social instability stress, which consists of alternating housing conditions for the animals, or chronic-mild stress, in which experimental rodents are exposed to unpredictable social and non-social stressors for several weeks [25]. However, there are no systematic reviews on preclinical models that have tried to assess the role of social stress and dominance on different aspects of inhibitory control deficit, which could help us to improve our knowledge in its vulnerability, prevention and treatment.

Therefore, the present review’s aim is to evaluate the possible influence of social stress and dominance on the vulnerability to develop a deficit inhibitory control, through the information provided by preclinical models.

## 2. Materials and Methods

An extensive literature search was conducted in November–December 2020 using electronic databases, including PubMed and Web of Science. The search keywords used were: social dominance, social isolation, impulsivity, impulsive behaviour, inhibitory control, compulsivity, compulsive behaviour, compulsive personality disorder, obsessive-compulsive disorder, OCD, TOC, binge drinking, alcohol drinking, drinking behaviour, animal models and rat. The Boolean operator “and” was used. No date limit was set to select a study. 

As for the inclusion criteria, articles written in English were included, and articles that obtained information about the relationship between social dominance and/or social stress and vulnerability to impulsive and/or compulsive spectrum disorder. Regarding the exclusion criteria, articles written in languages other than English were discarded and those not relevant to the main topic.

**Figure 1:**
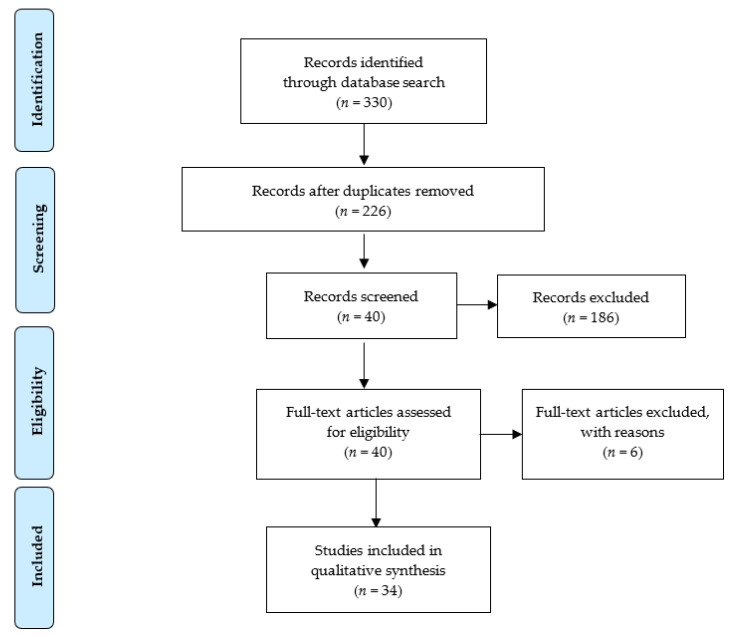
PRISMA flow diagram of search history and selection of articles.

Additionally, a search for “grey literature” was carried out using two databases: Open Grey and WorldCat. The same combinations used in the other databases were used in both. However, due to the length of the present work, it was decided not to include such information. Once the search was completed, duplicated works were discarded. The selection protocol was based on the reading of the titles and abstracts, discarding those not relevant to the study topic. Figure 1 shows a flow diagram of the literature search strategy and the review process following PRISMA 2009 flow diagram rules.

## 3. Results

According to the literature found, we collated and organized the results on social stress and dominance in preclinical models focusing on aggressive impulsivity and inhibitory control deficit associated with impulsive and compulsive drug intake.

### 3.1. Social Stress Aggressive, Competition and Impulsive Behaviour

Table 1 shows the literature on preclinical models of stress and social dominance on impulsive aggressive behaviours and competition for resources. In 2012, Coppens et al. [26] assessed Roman high avoidance (RHA) and Roman low avoidance (RLA) rats with different coping styles and impulsivity phenotypes to study the effects of social defeat stress. In the RIT, RHA rats showed higher attack latency, higher offensive aggression and spent more time in social interaction, while RLA rats spent more time grooming. During a variable interval programme of reinforcement for obtaining food and during subsequent extinction, RHA rats made more lever presses than RLA rats. Therefore, RHA rats were more impulsive and more affected by social stress than RLA rats. In recent research [27], male wild-type Groningen (WTG) rats were subjected to social defeat stress during adolescence by RIT. When their behaviour in operating conditioning for food reinforcement was observed, no differences were found in the number of lever presses or rewards between the stressed rats and the controls. During the RIT, no differences were found in offensive behaviour, social exploration, social interaction, non-social exploration, immobility and grooming. However, in the control group, a correlation was found between impulsivity and offensive aggression that was not found in animals exposed to social defeat. Regarding the possible relationship between wild rats and albinos in water consumption, Boice found that rats with scars consumed more water compared to rats without scars and albinos. These results showed the altered emotionality and low social status of the scarred wild rats [28].

In mice, Yang and colleagues found that in C57BL/6J mice that were exposed to chronic unpredictable mild stress (CUMS), sucrose consumption was reduced in the Sucrose Preference Test (SPT), which evaluates the response of preference in the consumption of different concentrations of sucrose, as well as the number of crossing, “rearings” and mobility time in an open field test (OFT). In the RIT, the stressed mice were more aggressive and had lower attack latency, and in the Tube Test (TT), they won more times than the controls. Treatment with fluoxetine (18 mg/kg) reduced aggression in stressed mice, while clozapine (20 mg/kg) also reduced social dominance behaviour. In conclusion, mice subjected to CUMS showed high levels of aggression and dominance [29]. In another similar study, using Sabra (Wt) and subordinate (Sub) mice also found that exposure to CUMS reduced sucrose consumption in SPT, leading to the development of polydipsia in both Wt and Sub mice. In the OFT, the stressed mice showed less locomotor activity as well as reduced social exploration measured through the Three-Chamber Test (3-CT), which consists of an apparatus with three chambers separated by a metal mesh, an unknown male is placed in a side room while the opposite room is empty, the wire mesh is removed, and the time spent by the mouse on each chamber is recorded [30].

Jupp et al. [31] investigated whether the social status in Lister Hooded rats assessed through the RCT influenced the development, maintenance and re-establishment of cocaine addiction. They found that dominant rats drank for longer periods than subordinate rats during the competition phase for a highly palatable liquid. In the TT, the rats designated as dominant using the RCT won more encounters than the subordinate ones. As for the acquisition of self-administration behaviour, no social rank effects were observed, although dominant rats pressed the lever more often than subordinate ones. Additionally, differences were observed during the extinction and re-establishment of self-administration behaviour. The dominant rats showed higher locomotor activity in a novel environment compared to the subordinate ones. In the Light-dark box (L-DB) (a chamber divided into a dark and a light compartment), no differences were found between the two groups, although all the rats preferred the dark area. Finally, in the Five-choice-serial-reaction time task, there were also no significant differences between subordinates and dominants, although longer intervals between trials increased premature responses.

In a study, Woodall and colleagues evaluated the effects of the administration of 8-hydroxy-2-(di-n-propylamino)tetralin (8-OH-DPAT), a selective agonist of the serotonin 5-HT1A receptors, in dominant, intermediate and subordinate Lister-Hooded rats of each triad to assess its impact on the task of competition for condensed milk. Of the groups tested in the study, 80% established dominant hierarchies and competed for condensed milk. When the effects of 8-OH-DPAT administration on subordinate rats were evaluated before the social competition test, they found that they had increased their access to the drinking spout, whereas dominant rats treated with 8-OH-DPAT did not increase social competition. On the other hand, 8-O-HT-DPAT did not affect the amount of condensed milk consumed or locomotor activity in dominant, intermediate and subordinate rats [32].

Lucion and Vogel subjected Sprague-Dawley rats to different types of stress to evaluate their performance in the Water Competition Test [33]. The situation of competition for water quickly established the dominant–subordinate relationship, which remained stable over time. A mild stressor (2 h) decreased aggressive behaviour (not significantly) and the differences in water intake between dominants and subordinates. Severe stress (18 h) reversed the dominant–submissive relationship in most pairs. When the severe stress was removed, eventually, the original relationship was restored. In the case of submissive rats, severe stress decreased aggressive behaviour, although not significantly. Anxiety before stress exposure caused anxious, dominant rats to lose more weight and more aggressive behaviour than low-anxious rats.

As the above suggests, animals exposed to social stress during adolescence may not present the same behavioural consequences. However, in RHA rats, social stress during adolescence appears to affect the levels of impulsivity and aggressive behaviour more than in RLA rats. Stress also affects mice by increasing levels of aggression and social dominance and can lead to the development of polydipsia in subordinate mice. In RCT, dominant rats tend to consume more food or fluid than subordinate rats. In the case of self-administration of cocaine, dominant rats press the lever more frequently than subordinate rats to obtain the drug, although no differences in impulsiveness are observed. Finally, when a dominant animal is subjected to severe stress, it is possible to reverse the dominant–submissive relationship even though it is a temporary phenomenon.

### 3.2. Social Stress, Drug Addiction and Impulsive Behaviour 

#### 3.2.1. Social Defeat and Psychostimulants Drugs

Table 2 shows preclinical models of stress induced by social defeat and its relationship with psychostimulants drugs. In one study [34], exposure before social defeat favoured locomotor response to heroin (0.03 mg/kg/infusion), cocaine (10 mg/kg) and speedball (a combination of heroin and cocaine) in Long-Evans rats. However, there were no differences in the acquisition and maintenance of self-administration between the group exposed to social defeat and the control group. During administration, according to a fixed ratio (RF) and progressive ratio (PR) reinforcement program, there were no differences between the two groups in the number of cocaine, heroin or speedball infusions. In contrast, by self-administration of “speedball” during a 24-h unlimited access binge, the rats exposed to social defeat showed a higher persistence in self-administration behaviour and a trend for escalated intake, especially in the last 12 h. Therefore, rats exposed to stress have a higher preference for cocaine consumption unless a heroin–cocaine combination is available. 

In a similar study [35], Long-Evans rats sensitized to stress, cocaine and morphine increased their locomotor responses. A cocaine dose of 7.5 mg/kg increased locomotion in rats previously treated with cocaine, while this dose did not increase locomotion in rats previously treated with morphine. Furthermore, in a PR schedule, cocaine sensitization facilitated the acquisition of cocaine self-administration and an increase in breaking points, which is defined as the last infusion administered in the session. During a 24-h unlimited access binge, cocaine- and stress-sensitized rats self-administered significantly more cocaine than controls. Likewise, these rats also maintained their self-administration behaviour for longer, thus accumulating a higher amount of drug. Non-sensitized animals stopped their consumption after approximately 18–20 h. 

Subsequent research [36] examined the relationship between access conditions to cocaine administration and a history of defeat stress in Long-Evans rats. On the one hand, they found that rats with prolonged access (6 h/day) to cocaine self-administered more infusions than short-access (1 h/day) animals in the first hour of the session, which maintained a similar intake during the 14 days of the experiment. The prolonged access condition favoured faster cocaine self-administration but did not affect the self-administration of the control group. On the other hand, the rats exposed to social defeat showed cross-sensitization to cocaine when compared to the control group, that is, previously stressed animals, showed an increased locomotor response to a subsequent challenge with a psychostimulant. In a PR schedule of reinforcement, socially defeated rats completed higher ratios of responding to obtain cocaine infusions and increased break points, as well as during a 24-h unlimited access binge. However, the prolonged access condition did not increase cocaine intake in non-stressed controls. As for mice, Yap and Miczek found that social defeat and amphetamine in CFW mice were sufficient to cause behavioural cross-sensitization to the stimulant effects of amphetamine. Amphetamine injections caused increased locomotor activity and a sensitized response to the lower dose of amphetamine compared to controls. Amphetamine pretreated mice exhibited increased cocaine self-administration during acquisition compared to the control group, as well as elevated break points during a PR schedule of reinforcement. However, exposure to social defeat did not increase cocaine self-administration in CFW mice, contrary to what is found in stressed rats [37].

Taken together, social defeat stress encourages cocaine use in stressed rats. Rats sensitized to stress or cocaine tend to develop an increase in cocaine use and make a higher number of responses to obtain the drug. However, exposure to social defeat stress does not appear to increase cocaine self-administration in CFW mice.

#### 3.2.2. Social Stress and Depressant Drugs

Table 3 shows the literature on preclinical models of stress and social dominance and its relationships with depressant drugs. Regarding the relationship between stress and alcohol consumption, Marcolin et al. [38] found that Long-Evans rats under social instability stress (daily 1 h of isolation after which they are housed with a new cage partner from the postnatal day) consumed more ethanol than non-stressed rats regardless of social context, that is, whether they were housed alone, with an unfamiliar peer or with a familiar mate. When comparing adolescent rats with adult rats in a Drinking Competition Task, they found that stressed adolescent but non-adult rats consumed more ethanol than controls. As for sucrose consumption, there were no differences between stressed rats and both adolescent and adult controls, except in adulthood under conditions of competition where stressed adolescent rats consumed more sucrose than controls and adults. In general, adolescent stressed rats housed alone or with a familiar partner had lower drinking latency and consumed more ethanol than controls. In recent research [39], using Intermittent Access to 2-bottle choice (2-BC) in which Long-Evans rats had access to a bottle of water and another bottle of water or 10% ethanol sweetened with 0.1% saccharin, no differences were found between rats exposed to social instability stress and control rats. In another condition, saccharin was added at 0.1% without ethanol, which caused a reduction in the consumption of stressed rats compared to control rats. Therefore, in this study, it could not be stated that exposure to stress during adolescence leads to a predisposition for ethanol consumption in adulthood. 

Another study [40] assessed whether chronic social instability would lead to increased consumption and preference for ethanol in Long-Evans rats. The main result was that stressed male rats had a higher preference for ethanol than control rats during weeks 2 and 3 of consumption, while water consumption in those weeks was lower compared to the other groups. Nevertheless, in female rats exposed to chronic social instability, there were no significant differences in ethanol consumption and preference. Regarding the possible relationship between duration of stress and ethanol consumption, van Erp et al. [41] found that Long-Evans rats exposed to short (30 m), intermediate (6 h) and continuous (24 h) stress decreased their ethanol consumption by 10% ethanol during the 5 days of stress exposure, while it did not affect the 3% ethanol solution and even increased their water consumption. As for the possible relationship between social dominance and drug use, Blanchard et al. [42] found that subordinate Long-Evans rats consumed more ethanol than dominant rats. Subordinate male rats licked more than dominant rats, with the frequency of licking being lower in the 8% ethanol condition than in the 4% condition. However, female rats had a higher licking frequency than males in the 4% ethanol condition, but there were no differences in the 8% ethanol condition. As for offensive behaviour, dominant male rats showed a decline when pre-ethanol consumption measures were compared with those taken during 8% ethanol administration. Female rats showed low frequency both before and during ethanol consumption in offensive and defensive behaviour. One study [43] found that subordinate Long-Evans rats consumed more ethanol than dominant and subdominant rats and rats housed in aggressive triads also consumed more ethanol than rats housed in non-aggressive triads. The OFT had little effect on ethanol consumption since it only decreased the consumption of subdominants in non-aggressive triads and did not affect ethanol preference either. The Elevated plus maze test decreased ethanol consumption and ethanol preference in subdominants regardless of the aggressiveness of the triad. Finally, after the RIT, ethanol consumption increased in all rats of non-aggressive triads, while it decreased in subdominants of aggressive triads. Ethanol preference also increased in dominant and subordinate rats. In one study [44], it was found that high-drinking rats scored low on several measures of dominance compared to low-drinking rats. In fights, high consumers lost more matches, rarely chased other rats and initiated less aggressive behaviours such as violent fighting and broadsiding, where the dominant animal shows the larger side of its profile to impress its opponents. High consumers also spent more time in the burrows and less time running on the wheel, although they were more engaged in “grooming” (cleaning activities and orofacial movements indicative of compulsivity) than low consumers. When alcohol was removed from the colony, the high consumers became more active and increased social grooming and chasing, while they did not increase in dominance. In recent research [45], it was found that the forced alcoholization model increased ethanol preference in subdominant and subordinate but not dominant Wistar rats. The same effect was achieved in the case of the group where only water was available. Furthermore, alcohol consumption in the 2-BC was maintained at a level of 10% in the rats that were group-housed even though they had no preference for alcohol. However, the rats housed alone after two months of forced alcoholization drank only water. 

In the case of mice, Bahi found that chronic psychosocial stress in C57BL/6 mice from the chronic subordinate colony compared to individually housed control mice increased ethanol consumption and preference in the 2-BC. However, no differences were found in the consumption and preference of sucrose and quinine between both groups [46]. Hilakivi-Clarke & Lister found that, before group housing, severely injured mice consumed more ethanol than mildly injured or alphas. During RIT, there were no differences in the aggressiveness exhibited by the three groups of mice. When mice were housed in groups, alcohol consumption was higher in submissive mice than in alpha or control mice. The preference for alcohol among the groups was the same, while the amount of total fluid was higher in submissive mice than in alpha and controls [47]. Some authors [48] found that continued exposure to defeat in C57 mice increased ethanol consumption, while the experience of victory decreased ethanol consumption and increased water consumption. However, victory and defeat did not affect ethanol consumption in CBA mice. In OFT, in the exploratory activity test and in the Forced Swimming Test (FST), which measures escape, immobility and gentle swimming behaviour, ethanol did not cause any behavioural changes in submissive mice as the number of nose pushes (pushes with the rat’s nose towards a bottle), rears (lifting of the front legs in an exploratory attitude) and immobility time, which are indicative of compulsivity, were similar in the losers who drank ethanol and water. In the hot-plate pain test, which consists of a hot plate where the animal is placed and measures the latency it takes to lick its paws or jump to avoid pain, the paw-lick latency (which may be indicative of compulsivity) was not affected by ethanol. Nevertheless, ethanol increased locomotor activity near the partition in submissive in the presence of the aggressive mouse in the Partition Test (PT), which assesses the social interest in a partner.

Wolffgramm and Heyne [49] found that ethanol consumption varied according to housing conditions (group housing, individual housing and contact caging). Long-term isolated rats consumed more ethanol and preferred a higher concentrated solution. When housing conditions were changed from a lower to a higher level of deprivation, ethanol consumption increased. However, in the opposite case, consumption levels remained constant. When ethanol was withdrawn and diazepam was offered, there were no differences in the choice of the latter. Additionally, dominant rats consumed significantly less than subordinate rats, but the former increased their ethanol consumption when housing conditions changed. Likewise, after a period of abstinence, rats with previous experience increased their ethanol consumption compared to their previous consumption. In mice, Kudryavtseva et al. [50] found that losers mice consumed more ethanol than the winner mice. After a deprivation period, the winners treated with placebo increased ethanol consumption, while the losers who were treated with U-50,488H, which has analgesic, diuretic and antitussive effects, consumed more ethanol than those treated with placebo. Furthermore, both after a period of deprivation and after ethanol consumption, the performance in the PT of the winners (regardless of whether they consumed ethanol or not) continued to be one of the frequent approaches to the partition while the losers approached less often and for less time, indicating that the losers have a less social interest and greater anxiety. However, U-50,488H increased the number of times the losers approached the partition while it decreased the approach of the winners. One study [51] evaluated the consequences of defeat stress in NMRI mice. Subordinate mice during the PT approached the partition less frequently than dominant mice. When their ethanol consumption was examined, no differences were found in the amount consumed compared to the controls. No significant differences were found between groups in the FST. During the L-DB test, the subordinates spent less time in the illuminated section than the controls and showed less exploratory activity. However, when citalopram was administered (20 mg/kg/day), the subordinates increased the time spent in the illuminated section and increased the exploration.

In an investigation, Duncan et al. [52] used the visible burrow system (VBS), which consists of an area with an open surface according to a 12 h light/dark cycle, with two smaller covered chambers connected by covered tunnels. The small chambers and tunnels are always in darkness. Water and food are always available on the open surface area and in the small chambers. It was found that rats treated with alcohol failed to establish a hierarchy, unlike the group treated with sucrose. Additionally, the alcohol-treated rats decreased their alcohol consumption in VBS compared to the baseline, and there was no effect of social status on consumption during VBS in the group that received alcohol and sucrose. Rats treated with alcohol showed less offensive behaviour and fewer bites compared to the group treated with sucrose while housed at VBS. In another study [53], it was found that Long-Evans rats designated as dominant through agonist encounters consumed less ethanol than subdominant rats in the pre-dyad test. However, during the dyad test, the dominant rats consumed more ethanol than the subdominant and individually housed rats, which decreased their consumption levels or maintained them, although this effect was not statistically significant.

Regarding the possible relationship between stress and drug use, Heyne found that by housing Wistar rats in different conditions (group caging, single caging and contact caging), the preference and intake of the opiate etonitazene (ETZ) varied. In particular, single housed rats or contact caging consumed more opiates than rats housed in groups. Furthermore, in rats where ETZ consumption was forced, intake was higher than in the control group, and in dominant rats, consumption was significantly lower than in subordinate rats. By changing the housing conditions after 10 weeks, ETZ intake was adjusted to each condition so that rats previously group-housed and later socially deprived increased their ETZ consumption and vice versa. Additionally, during the last 5 weeks of opiate access, all rats increased their voluntary intake of ETZ. After a period of abstinence, socially deprived rats increased their consumption of ETZ compared to their previous consumption [54]. As for the possible relationship of fluoxetine with social status, Mongillo et al. [55] found a differential effect of fluoxetine in naked mole-rats according to status (subordinates and queen) and social environment (in-colony and in a social-pairing paradigm). Subordinates in-colony treated with fluoxetine spent less time digging and climbing, and in general, both the fluoxetine-treated and the controls showed little aggressive behaviour. When subordinates were subjected to the out-pairing paradigm, fluoxetine-treated animals had a lower frequency of aggression than controls. However, when they were paired with soldiers instead of workers, they increased the duration of the aggression. Concerning the queens, fluoxetine decreased sniffing behaviour and did exhibit aggression in-colony, in contrast to subordinates. Nevertheless, in the out-pairing paradigm, fluoxetine treatment significantly reduced aggressive behaviour in queens as well as its duration when directed at soldiers. In another study, Endo et al. [56] evaluated the impact of the administration of 2,3,7,8-tetrachlorodibenzo-p-dioxin (TCDD), a toxic of the dioxin family that induces abnormal sexual dysmorphic behaviour in adulthood. To this end, doses of 0, 0.3 and 0.6 μg/kg were orally administered to pregnant mice of the C57BL/6 strain, and the behaviour of the offspring was subsequently evaluated. TCDD did not affect the exploratory activity and motor coordination during the acclimation phase of the behavioural flexibility test, the OFT and the Rota Rod Test. However, TCDD caused the mice to take longer to achieve a behavioural shift in the serial reversal task. During the stay of the mice in the IntelliCage, an automated behavioural test apparatus for mice under a group-housed condition that was used to analyse the competitive domain in limited access to water, the mice called TC-0.6 because they were exposed to the dose of 0.6 μg/kg of the mentioned compound showed a deficit in the executive functions since they exhibited compulsive, repetitive nose poking as opposed to the group TC-0.3 exposed to the intermediate dose. TCDD affected the competitive dominance of TC-0.6 mice as they visited the corners of the IntelliCage less often to obtain water in the presence of another group, but not when each group was housed separately. The behavioural alterations observed induced by TCDD in the TC-0.6 group could be due to hypoactivation in the medial prefrontal cortex (mPFC) and hyperactivation of the amygdala.

From the above, it could be concluded that, on the one hand, stress may trigger a higher subsequent consumption of alcohol or opiates through different housing conditions. On the other hand, a longer duration of stress seems to decrease the preference for a more concentrated ethanol solution. However, there are contradictions as to whether or not exposure to stress necessarily leads to increased ethanol consumption. Alcohol consumption can favour the reduction of aggressiveness and prevent the formation of hierarchies. Furthermore, subordinate rats generally consume more alcohol than dominant rats and score low on several measures of dominance. Additionally, rats housed in aggressive triads tend to consume more alcohol than rats housed in non-aggressive triads. As with rats, subordinate mice also tend to consume more alcohol than dominant mice. The administration of U-50,488H favours alcohol consumption in loser mice; however, C57 mice are more vulnerable to the effects of social defeat on alcohol consumption than CBA mice. Moreover, fluoxetine affects behaviour according to status and social context and is effective in decreasing aggression in dominant male rats when paired. On the other hand, perinatal exposure to TCDD seems to deteriorate behavioural flexibility, decrease competitive dominance and produce compulsive, repetitive behaviour.

### 3.3. Social Stress and Dopaminergic Mechanisms

Table 4 shows the literature regarding the possible relationship between exposure to social stress and dopaminergic D1/D2 receptors. Burke et al. [57] found, on the one hand, that non-stressed amphetamine-treated rats had lower levels of expression of the dopaminergic D2 receptor protein in the NAc than non-stressed saline-treated rats, while stressed rats had higher levels of the dopaminergic D2 receptor compared to amphetamine-treated controls. Rats that had been treated with a saline solution and that belonged to the social defeat group or the control group showed no differences in the expression of the dopaminergic D2 receptor. The social defeat also did not alter the expression of the dopaminergic D2 receptor in the mPFC, the NAc shell and the striatum. Furthermore, rats exposed to social defeat during adolescence and subsequent amphetamine conditioning in adulthood did not result in the appearance of alterations in the expression of dopaminergic D1 receptors in any area of the brain. On the other hand, rats that were exposed to foot-shock and treated with saline in adolescence also had lower dopaminergic D1 receptor levels in the striatum compared to the non-stressed saline group. Thus, exposure to stress from social defeat but not from exposure to foot-shock prevents the down-regulation of dopaminergic D2 receptors in NAc by amphetamine. Regarding the relationship of social dominance with dopaminergic D2/D3 receptors [31], it was found that in rats designated as dominant after the RCT, the expression of the dopaminergic D2/D3 receptors was significantly higher in the dorsal striatum and the NAc shell compared to subordinate rats. Dopamine transporter (DAT) binding was also found to be higher in the NAc shell of dominant rats. No differences were found in serotonin transporter (5-HHT) binding. However, dominant rats had lower levels of dopamine (DA) in the NAc shell compared to subordinate rats. In a recent study in which saline or the antagonist SCH23390 were administered to block the dopaminergic D1 receptor in mice, it was found that SCH facilitated social dominance (measured by TT) in mice at the middle, but not high or low, social rank in the groups. In the 3-CT test, the mice that received only saline spent more time in the social area than in the non-social area, while mice treated with only SCH decreased the social preference of the mice. However, mice treated with both SCH and saline also spent more time in the social area. In mice in pairs, high and low range, SCH did not affect the time spent in the social area. In the radial 8-arm maze test, both the number of times the mice entered the same arm at least twice non-consecutive (NC) and the consecutive entries in the same arm (C) were recorded to evaluate working memory and behavioural flexibility. On the one hand, the mice that received SCH increased the number of NC and C entries, the first-rank mice performing the worst. On the other hand, mice administered with SCH were more likely to enter the open arms, with the first-rank mice exhibiting the most impulsive behaviour [58].

In conclusion, stress experienced in adolescence may alter how amphetamine exposure in adulthood down-regulates the dopaminergic D2 receptors. Social dominance appears to cause changes in the striatal dopamine system and variations in the dopaminergic D2 receptor in the striatum. Besides, social dominance in group-housed mice also appears to be facilitated by low dopaminergic D1 receptor functions.

## 4. Discussion

The present review constitutes a summary of the main studies found regarding the role of social dominance and stress as predisposing factors for the development of an impulsive phenotype, excessive drug use and the possible involvement of dopaminergic D1/D2 receptors in the development of addictive behaviour. Therefore, we will address the relationship between social dominance and stress with abusive consumption and aggressive, impulsive behaviours, and finally, we will discuss the involvement of dopaminergic receptors in the development of these behaviours. The main results regarding the role of social dominance and stress as predisposing factors for the development of an impulsive phenotype, excessive drug use and the possible involvement of dopaminergic D1/D2 receptors in the development of addictive behaviour are presented below.

### 4.1. Role of Dominance and Social Stress as Factors of Vulnerability to the Development of an Impulsive Phenotype

Thanks to this review, we have been able to better understand the involvement of dominance and social stress in the development of an impulsive phenotype, a vulnerability factor for the development of addictive behaviours [59,60,61]. On the one hand, we have found publications that relate the experience of socially stressful and submissive situations to an increase in impulsive and aggressive behaviour [62]. On the other hand, we have observed that different authors show that the presence of aggressive and impulsive behaviours increases the risk of suffering from addiction to some types of substances [63].

The review of studies shows similar effects of social dominance and stress on the development of aggressive, impulsive behaviour. In the case of RHA and RLA rats, one study found that RHA rats showed higher offensive aggression, higher impulsivity and higher vulnerability to the effects of social defeat stress [26]. In contrast, another study found no correlation between impulsivity and offensive aggression in rats exposed to social defeat [27]. In the case of mice, it has been observed that stressed mice are more aggressive than those not exposed to stress [29]. Subordination stress has also been found to lead to the development of polydipsia in subordinate mice [30] and increased water consumption in subordinate rats [28]. 

Several animal studies support the idea that social dominance is a key factor in the development of an impulsive phenotype and addiction. Aggressiveness leads to the development of impulsive behaviour in hamsters in the delay discounting paradigm [64] and rats when they are subjected to a variable interval of reinforcement during operant conditioning [65]. Aggressiveness also increases cocaine and morphine self-administration in mice and develops a conditioned preference for morphine [66]. In socially dominant monkeys, cocaine use is lower than in subordinate monkeys [67], and dominant female monkeys are more vulnerable to the reinforcing effects of cocaine than subordinate ones [68]. In human research, social dominance has been found to regulate the interaction between testosterone and the risk of developing a substance use disorder, being individuals characterized by social dominance and a tendency to violate social norms those who have the highest risk of developing addiction [69].

As for the relationship between social stress and addictive behaviour, exposure to social defeat by stress also induces behavioural sensitisation to a subsequent amphetamine challenge in rats [70]. Furthermore, stress increases the rate of acquisition of heroin self-administration in rats [71]. Additionally, exposure to social stress during adolescence leads to increased cocaine self-administration and cocaine-induced conditioned place preference in mice [72,73]. In several studies, exposure to subchronic and mild social defeat stress increased water consumption in mice with aspects similar to polydipsia [74,75]. In research with prisoners, it has been found that stressors such as problems with friends and family are associated with drug use 30 days after release from prison [76]. Exposure to stress during childhood in children is also a risk factor for the development of a substance abuse disorder [77]. 

In summary, a large number of studies have proven that both social stress and stress due to subordination facilitate the appearance of aggressive behaviour, the increase in impulsive behaviour such as self-administration of drugs in both animals and humans, and the appearance of polydipsia which is related to compulsivity. For this reason, it could be affirmed that situations of social stress and subordination seem to be related to impulsive and compulsive behaviours.

### 4.2. Excessive Consumption in Rodents under Social Stress

Most of the studies found agree that exposure to stress through repeated social defeat increases drug use in rats, as well as persistence in self-administration behaviour compared to non-stressed rats. Additionally, stress increases the locomotor response of rats to the presence of drugs such as cocaine, heroin and morphine [34,35,36]. However, social stress seems not to affect mice in the same way as it does not increase self-administration behaviour [37]. Similarly, most of the studies found also support the idea that exposure in rats to social stress leads to a higher preference and consumption of alcohol than in non-stressed rats [38,49] with some exceptions [39,40]. One study even confirms that longer or shorter duration of stress affects the consumption of different ethanol solutions [41]. In addition, the research confirms that housing conditions can affect the consumption of alcohol and opiates, with rats housed alone consuming the highest amount of drugs [49,54]. On the other hand, many studies show that subordinate and more defeated rats consume more and have a higher preference for alcohol than dominant rats [42,43,45,47,49,53]. This phenomenon also occurs in the case of mice [46,47,48]. Further, Duncan et al. [52] found that alcohol complicates the formation of hierarchies and reduces offensive behaviour in rats.

Studies with humans have found a relationship between alcohol consumption and social problems such as arguments and absenteeism from work [78].

Some studies confirm that exposure to fluoxetine, which is a selective inhibitor of serotonin reuptake in the presynaptic membrane, decreases aggressive behaviour in dominant mole-rats [55]. This same result has been found in other species such as coral reef fish [79], long-finned zebrafish [80] and Betta splendens [81]. However, other studies indicate that fluoxetine has no effect on aggressiveness in striped shore crabs [82], and even that fluoxetine increases aggressiveness in species such as Brycon amazonicus fish [83] and hamsters [84]. In humans, treatment with fluoxetine is effective in reducing symptoms of obsessive-compulsive disorder in adults, adolescents and children [85,86,87]. On the other hand, fluoxetine has been demonstrated to be effective in reducing ethanol consumption in female rats [88] and rats with a high preference for alcohol [89]. In humans, it has also been effective in reducing alcohol consumption in adults [90]. Exposure to TCDD, a toxic of the dioxin family, in addition to producing behavioural inflexibility, decreases competitive dominance and produces compulsive, repetitive behaviour in dose-dependent mice [56]. In a study with rats, exposure to a low dose of TCDD (200 ng/kg) resulted in altered paired-associate learning [91]. In another investigation, TCDD (0.18 and 0.54 μg/kg) affected performance in a random ratio and delayed alternation test in rats [92]. Regarding alcohol consumption, TCDD seems not to affect alcohol consumption in mice with high and low alcohol preference [93]. In humans, prenatal exposure to polychlorinated biphenyls, some of which are in the dioxin family, appears to increase the risk of becoming tobacco and alcohol users [94]. 

On the other hand, some studies show that when rats are exposed to an RCT, the dominant–submissive relationship is often established quickly, and dominant rats consume more fluid or food than subordinate rats [31]. This effect can be modulated by the administration of drugs such as 8-OH-DPAT [32]. This k-opioid receptor agonist has been found to reduce consumption in rats with simulated injuries and a high preference for alcohol [95]. In addition, increasing alcohol consumption in low doses and decreasing it in high doses in rats and squirrel monkeys (Saimiri sciureus) depending on the dose [96,97]. In humans, administration of morphine [98] and codeine [99] have been found to increase aggressive behaviour.

It could be highlighted that several studies have shown how situations of social stress and subordination favour the increase of drug and alcohol consumption in different species. Furthermore, treatment with certain substances such as fluoxetine and 8-OH-DPAT has been shown to have a therapeutic effect, decreasing aggressive behaviours and, in turn, compulsive and addictive behaviours. Thus, we could affirm that aggressiveness and compulsion seem to be correlated behaviours.

### 4.3. Possible Involvement of Dopaminergic Receptors D1/D2 in the Development of Addictive Behaviour

Dopamine is the crucial reward neurotransmitter in the brain activated by drugs of abuse [100]. The receptors of this neurotransmitter, which are divided into two families: the dopaminergic D1/D2 receptors, related to motivational and reward processes [101], have a primary role in the development and maintenance of impulsive and addictive behaviours.

On the one hand, regarding the relationship of social defeat stress with dopaminergic D1/D2 receptors, it has been found that exposure to defeat stress during adolescence prevents the regulation of dopaminergic D2 receptors in NAc by amphetamine in adulthood, while such exposure does not seem to affect dopaminergic D1 receptors [57]. On the other hand, regarding the relationship with social dominance, it has been found that dominant rats have greater expression of the dopaminergic D2 receptor in the dorsal striatum and the NAc shell compared to subordinate rats. Furthermore, the binding of the dopamine transporter (DAT) is also higher in the NAc shell of the dominant ones, although they have lower levels of DA in the NAc shell than the subordinate ones [31]. The use of the antagonist SCH23390 to block the dopaminergic D1 receptor in mice facilitates social dominance in second rank mice, decreases social preference and favours impulsive behaviour in first rank mice [58].

In studies with rats, it has been found that the activation of the D1/D2 heteromer nullifies cocaine-conditioned place preference, locomotor sensitization, self-administration and re-establishment of cocaine-seeking, being fundamental to control the development of cocaine addiction [102]. Additionally, rats with high rates of cocaine use are less affected by dopaminergic D1 receptor agonists such as SKF 81297, which inhibit cocaine seeking. However, a dopaminergic D2 receptor agonist, such as quinpirole, which stimulates cocaine seeking, affects high-user rats more [103]. In one study in rats, while quinpirole decreased alcohol consumption, spiperone (D2-antagonist) increased or decreased alcohol consumption depending on the dose. When the D1 agonist SKF-3839 was administered, alcohol consumption decreased, while when the D1 antagonist SCH-23390 was administered, consumption decreased according to the dose administered [104]. In a recent investigation where shRNA (a lentiviral construct containing a short hairpin RNA) was used to block the expression of dopaminergic D1 receptors in the dorsal striatum, rats were found to have no alteration of dopaminergic D2 receptors, increased their response rate and consumed more amphetamine than rats in which shRNA was not used [105]. 

In people with stimulant use disorder, the administration of pramipexole, a dopamine agonist, reverses perseverative responses while it does not affect people with OCD [106]. In another study, the administration of pramipexole and amisulpride, a dopaminergic D2 receptor antagonist, reduced persistent responses in people with stimulant use disorder [107]. Naltrexone, a non-selective opioid antagonist, is effective in the treatment of alcohol-related problems, such as withdrawal and relapse [108], and in reducing craving [109]. However, another opioid receptor antagonist, naloxone, aggravates the symptoms of OCD [110]. 

In conclusion, several studies show that both stress and social dominance cause alterations in the dopaminergic D1/D2 receptors and that the administration of agonists and antagonists of these receptors can favour impulsive behaviours such as the consumption of alcohol and drugs such as cocaine and amphetamine. Therefore, we can say that the alterations caused in the AD receptors by social stress and dominance may be predisposing factors for the development of an impulse spectrum disorder.

## 5. Limitations and Future Lines

There are several limitations from the reviewed studies to understand the association and contribution of social stress and/or social dominance in the development of inhibitory control disorders. Most of the studies on social stress and/or social dominance are focused on certain aspects of inhibitory control, thus on the development of addiction and alterations in aggressive behaviour. More research is needed in terms of the possible effects of social stress and/or social dominance in different facets of impulsive and compulsive behaviours, such as cognitive flexibility, decision making or motor impulsivity. Moreover, the possible differences between both sexes in terms of the effect of social stress and development of inhibitory control deficit have not been extensively studied, as most of the experiments were in male subjects. Finally, another limitation is that the present review collates articles that were published and written in English. This may have excluded articles that were published in other languages. In future research, for a better knowledge of the implication of social stress and/or social dominance in inhibitory control deficit, it would be convenient to perform studies that: include specific tasks based on the different facets of impulsivity and compulsivity, compare the effect of chronic versus acute stress, as well as the effects in both sexes. Finally, a review that could collate the relationship between social dominance and the development of behavioural alterations related to impulsivity and compulsivity in humans could help understand its implication with a translational approach.

## 6. Conclusions

The present review allows us to conclude that both stress and social dominance can induce behavioural alterations related to inhibitory control deficit in rats and mice. Some of these alterations include (1) alterations in aggressive behaviour, (2) consumption of larger amounts of drugs, including alcohol, and (3) alterations in dopaminergic D1/D2 receptors.

This review aimed to test whether dominance or social stress can influence the development of an impulsive and/or compulsive spectrum disorder. In conclusion, social stress and dominance appear to be predisposing factors in the development of such disorders. Several studies support the idea that both social stress and subordination appear to be related to impulsive and compulsive behaviours such as the consumption of alcohol, drugs and the development of polydipsia. Additionally, a correlation has been found between aggressive behaviour and compulsion, and alteration of dopaminergic receptors caused by social stress and dominance also plays a role in the development of addiction. In summary, most of the studies show that both subordination and exposure to social stress increase drug use, social stress increases levels of aggression and both stress and social dominance cause alterations in the dopaminergic D1/D2 receptors.

## Figures and Tables

**Table 1:** Social stress aggressive, competition and impulsive behaviour.

Animal	Strain & Sex	Model	Test	Drug	Effect	Reference
Rat	Norway M				↑ Water intake in rats with scar-markings	Boice R. 1971 [28]
Rat	S-DM	Restriction stress (mild = 2 h, severe = 18 h)	OFT WCT		Severe stress inverted transiently the DOM-SUB relationship ↓ Aggressive behavior in anxious rats	Lucion A. & Vogel W. 1994 [33]
Rat	L-H M	Social competition	RIT	8-OH-DPAT (25 and 37.5 μ/kg)	↑ Social competition in SUB but not in DOM	Woodall K. et al., 1996 [32]
Rat	RHA RLA M	Social defeat stress, social isolation	RIT OC (IV-15)		↑ Attack latency, offensive aggression and lever presses in RHA RHA are more impulsive than RLA	Coppens C. et al., 2012 [26]
Rat	W-TG M	Social defeat stress	RIT		No differences in aggression and impulsivity Control animals showed a correlation between offensive aggression and impulsivity	Coppens C. et al., 2014 [27]
Mouse	C57BL/6J M	Chronic unpredictable mild stress	RIT SPT OFT TT	Fluoxetine (18 mg/kg) Clozapine (20 mg/kg)	↓ Sucrose preference and locomotion ↑ Aggressiveness in stressed ↓ Aggressiveness after fluoxetine treatment Clozapine reversed the symptoms of the stress	Yang C. et al., 2015 [29]
Mouse	Sabra (Wt) M	Chronic unpredictable mild stress	SPT 3-CT OFT EPM	Sucrose (2.5%)	↓ Sucrose preference and social exploration ↑ Anxiety Polydipsia in SUB	Gross M. & Pinhasov A. 2016 [30]
Rat	L-H M	Social competition	RCT TT L-DB 5-CSRTT	Cocaine (0.1, 0.2 and 0.4 mg/kg)	DOM drink longer than SUB ↑ Locomotion in DOM No differences in impulsivity	Jupp B. et al., 2016 [31]

Abbreviations: S-D (Sprague-Dawley); L-H (Lister-Hooded); RHA (Roman high avoidance); RLA (Roman low avoidance); W-TG (wild-type Groningen); OFT (Open Field Test); WCT (Water Competition Test); RIT (Resident-intruder Test); OC (Operant Conditioning); SPT (Sucrose Preference Test); TT (Tube Test); 3-CH (Three-Chamber Test); EPM (Elevated Plus Maze); L-DB (Light-dark box); 5-CSRTT (Five-choice serial-reaction time task); 8-OH-DPAT (8-hydroxy-2-(di-npropylamino)tetralin).

**Table 2:** Social defeat and psychostimulants drugs.

Animal	Strain & Sex	Model	Test	Drug	Effect	Reference
Rat	L-E M	Social defeat stress	RIT	D-Amphetamine (1 mg/kg) Cocaine (7.5, 10 mg/kg)	↑ Locomotion, breaking points and cocaine self-administration ↑ Consumption in stressed during a 24-h binge	Covington H. & Miczek K. 2001 [35]
Mouse	CFW M	Social defeat stress	RIT	Amphetamine (1–2.5 mg/kg) Cocaine (0.3–1.18 mg/kg)	↑ Locomotion, amphetamine intake Stress does not increase cocaine intake	Yap J. & Miczek K. 2007 [37]
Rat	L-EH M	Social defeat stress	RIT	Cocaine (0.75 mg/kg)	↑ Locomotion, breaking points and intake of cocaine ↑ Intake during a 24-h unlimited access binge	Quadros I. & Miczek K. 2009 [36]
Rat	L-E M	Social defeat stress	RIT	Heroin (0.25 mg/mL) Cocaine (10 mg/mL) Speedball (0.08 mg/mL heroin and 1.67 mg/mL cocaine)/(0.17 mg/mL heroin and 1.76 mg/mL cocaine)	↑ Cocaine and Speedball intake Sensitized locomotor behaviour ↑ Consumption in stressed during a 24-h binge	Cruz F. et al., 2011 [34]

Abbreviations: L-E (Long-Evans); L-EH (Long-Evans hooded); RIT (Resident-intruder test).

**Table 3:** Social stress and depressant drugs.

Animal	Strain& Sex	Model	Test	Drug	Effect	Reference
Rat	L-E M	Alcoholism		EtOH (10%)	↓ Dominance in high drinkers ↑ Activity after EtOH withdrawal	Ellison G. et al., 1983 [44]
Rat	L-E M+H	Social stress		EtOH (4 and 8%)	↑ EtOH intake in SUB and in femalesEtOH (8%) ↓ offensive attacks in DOM males	Blanchard R. et al., 1987 [42]
Mouse	C57BL/6J CBA/Lac M	Social stress	OFT EAT FST H-PPT PT	EtOH (20%)	↑EtOH intake in C57BL/6J ↓ Water intake in C57BL/6JEtOH ↑ the time near the partition in DOM	Kudryavtseva N. et al., 1991 [48]
Rat	W M	Social isolation	OFT FCP	EtOH (5, 10 and 20%) Diazepam (50 and 100 mg/L)	↑ EtOH intake in isolated rats ↓ EtOH intake in DOM Rats with high EtOH consumption also prefer diazepam	Wolffgramm J. & Heyne A. 1991 [49]
Mouse	Swiss NIH M	Social stress	RIT	EtOH (5%)	↑ EtOH consumption in severely wounded submissive mice No differences in aggressive behaviour	Hilakiviclarke L. & Lister R. 1992 [47]
Rat	W M	Social isolation	2-BC	ETZ (2, 4 and 8 mg/L)	↑ Intake in isolated and contact caging rats ↓ Intake in DOM ↑ Intake after a deprivation period	Heyne A. 1996 [54]
Mouse	NMRI M	Social defeat stress, sensory contact model	2-BC FST L-DB	EtOH (96%) Citalopram (20 mg/kg)	No differences in EtOH consumption ↑ Exploration ↓ anxiety in SUB after Citalopram treatment	Keeney A. & Hogg S. 1999 [51]
Rat	L-E M	Short (60 m), intermediate (6 h) and continuous (24 h) social stress	RIT	EtOH (3 and 10%)	↓ Intake of 10% EtOH ↑ Water intake	van Erp A. et al., 2001 [41]
Mouse	C57BL/LJ M	Sensory contact model	2-BC PT	U-50,488H (2.5 mg/kg) EtOH (10%)	↑ EtOH intake in losers ↑ Visits to the partition in losers after EtOH and U-50,488H treatment	Kudryavtseva N. et al., 2006 [50]
Rat	L-E M	Social stress	VBS EPM	EtOH (0, 2.5, 5, 7.5 and 10%) Quinine (25–750 μM) Sucrose (0, 2.5, 5, 7.5 and 10%)	↑ EtOH intake in SUB Colonies treated with EtOH did not establish hierarchies and showed less aggressiveness	Duncan E. et al., 2006 [52]
Rat	W M	Forced alcoholization	RIT ST 2-BC	EtOH (10%)	↑ EtOH preference in SUB and SUBdom No differences in anxiety and locomotion	Filatovaa E. et al., 2010 [45]
Rat	L-E M	Social defeat stress	RIT OFT EPM	EtOH (6%)	↑ EtOH consumption in SUB ↑ EtOH consumption in aggressive triads ↓ EtOH consumption in SUBdom after EPM	Pohorecky L. 2010 [43]
Rat	L-E M	Social isolation	2-BC	EtOH (1–6%) Sucrose (0.1%) Amphetamine (0.3, 0.9 and 2.7 mg/kg)	↑ EtOH intake in DOM dyad-housed Amphetamine (2.7 mg/kg) suppressed EtOH intake	Pohorecky L. & Sweeny A. 2012 [53]
Mouse	C57BL/6 F	Prenatal stress	IC-T OFT RRT	TCDD (0, 0.3 and 0.6 mg/kg)	↓ Dominance Behavioural inflexibility and compulsive repetitive behaviour (nose poking)	Endo T. et al., 2012 [56]
Mouse	C57BL/6 M	Chronic psychosocial stress, social isolation	2-BC EPM OFT	EtOH (2.5, 5, 10 and 20%)	↑ Anxiety, EtOH intake and conditioned place preference	Bahi A. 2013 [46]
Rat	Naked mole-rat M+F	In-colony and social-pairing paradigm		Fluoxetin (10 mg/kg)	↓ Climbing and digging behaviour in Queens and SUB ↓ Sniffing and aggression in Queens	Mongillo D. et al., 2014 [55]
Rat	L-E M+F	Chronic social instability stress	EPM 2-BC	EtOH (20%)	↑ Anxiety and EtOH intake in male rats Stress does not affect female rats	Roeckner A. et al., 2017 [40]
Rat	L-E M	Social instability stress	RCT 2-BC	EtOH (1.10%) Sucrose (2.1%)	↑ EtOH intake in stressed rats and in adolescent rats ↑ Sucrose intake in stressed adolescent rats	Marcolin M. et al., 2019 [38]
Rat	L-E M	Social instability stress	EPM 2-BC SIT	EtOH (10%) Sucrose (0.1%)	No differences in EtOH consumption ↓ Social interaction and anxiety in stressed rats	Marcolin M. et al., 2020 [39]

Abbreviations: L-E (Long-Evans); W (Wistar); OFT (Open Field Test); EAT (Exploratory Activity Test); FST (Forced Swimming Test), H-PPT (Hot-plate Pain Test); PT (Partition Test); FCP (Free Choice Paradigm); RIT (Resident-intruder Test); 2-BC (2-Bottle Choice); L-DB (Light-dark box); VBS (Visible Burrow System); EPM (Elevated Plus Maze); ST (Suok Test); IC-T (IntelliCage Test); RRT (Rota Rod Test); RCT (Resource Competition Task); SIT (Social Interaction Test); EtOH (Ethanol); ETZ (Etonitazene); TCDD (2,3,7,8-tetrachlorodibenzo-p-dioxin).

**Table 4:** Social stress and dopaminergic system.

Animal	Strain & Sex	Model	Test	Drug	Effect	Reference
Rat	S-D M	Social defeat stress, Foot-shock	CPP	Amphetamine (1 mg/kg)	↑ D2 receptor protein levels after social defeat ↓ D1 levels after foot-shock	Burke A. et al., 2011 [57]
Rat	L-H M	Social competition	RCT TT L-DB 5-CSRTT	Cocaine (0.1, 0.2 and 0.4 mg/kg)	High expression of D2 in NAc Shell and striated and DAT in DOM ↓ Dopamine in NAc Shell in DOM	Jupp B. et al., 2016 [31]
Mouse	CD1 M		TT 3-CT R8-AM	SCH (0.1 mg/kg)	Facilitated social dominance in mice at the second rank ↓ Social preference and anxiety	Yamaguchi Y. et al., 2017 [58]

Abbreviations: S-D (Sprague-Dawley); L-H (Lister-Hooded); RCT (Resource Competition Task); TT (Tube Test); L-DB (Light-dark box); 5-CSRTT (Five-choice serial-reaction time task); CPP (Conditioned Place Preference): 3-CT (Three Chamber Test); R8-AM (Radial 8-Arm Maze); SCH (SCH23390).

## References

[1] Dalley J.W., Everitt B.J., Robbins T.W. (2011). Impulsivity, Compulsivity, and Top-Down Cognitive Control. Neuron.

[2] Robbins T.W., Gillan C.M., Smith D.G., de Wit S., Ersche K.D. (2012). Neurocognitive endophenotypes of impulsivity and compulsivity: Towards dimensional psychiatry. Trends Cogn. Sci..

[3] Robbins T.W., Vaghi M.M., Banca P. (2019). Obsessive-Compulsive Disorder: Puzzles and Prospects. Neuron.

[4] Fineberg N.A., Apergis-Schoute A.M., Vaghi M.M., Banca P., Gillan C.M., Voon V., Chamberlain S.R., Cinosi E., Reid J., Shahper S. (2018). Mapping Compulsivity in the DSM-5 Obsessive Compulsive and Related Disorders: Cognitive Domains, Neural Circuitry, and Treatment. Int. J. Neuropsychopharmacol..

[5] American Psychiatric Association (2013). Diagnostic and Statistical Manual of Mental Disorders.

[6] Anderson S.E., Keim S.A. (2016). Parent-Child Interaction, Self-Regulation, and Obesity Prevention in Early Childhood. Curr. Obes. Rep..

[7] Ma I., van Duijvenvoorde A., Scheres A. (2016). The interaction between reinforcement and inhibitory control in ADHD: A review and research guidelines. Clin. Psychol. Rev..

[8] Venniro M., Zhang M., Caprioli D., Hoots J.K., Golden S.A., Heins C., Morales M., Epstein D.H., Shaham Y. (2018). Volitional social interaction prevents drug addiction in rat models. Nat. Neurosci..

[9] Huppert J.D., Simpson H.B., Nissenson K.J., Liebowitz M.R., Foa E.B. (2009). Quality of life and functional impairment in obsessive-compulsive disorder: A comparison of patients with and without comorbidity, patients in remission, and healthy controls. Depress. Anxiety.

[10] Rodriguez-Salgado B., Dolengevich-Segal H., Arrojo-Romero M., Castelli-Candia P., Navio-Acosta M., Perez-Rodriguez M.M., Saiz-Ruiz J., Baca-Garcia E. (2006). Perceived quality of life in obsessive-compulsive disorder: Related factors. BMC Psychiatry.

[11] Moritz S., Wahl K., Ertle A., Jelinek L., Hauschildt M., Klinge R., Hand I. (2009). Neither saints nor wolves in disguise: Ambivalent interpersonal attitudes and behaviors in obsessive-compulsive disorder. Behav. Modif..

[12] Moritz S., Niemeyer H., Hottenrott B., Schilling L., Spitzer C. (2013). Interpersonal Ambivalence in Obsessive-Compulsive Disorder. Behav. Cogn. Psychother..

[13] Johnson T.J., Sheets V.L. (2004). Measuring College Students’ Motives for Playing Drinking Games. Psychol. Addict. Behav..

[14] Radimer S., Rowan-Kenyon H. (2019). Undergraduate Men’s Alcohol Consumption: Masculine Norms, Ethnic Identity, and Social Dominance Orientation. J. Coll. Stud. Dev..

[15] Good G.E., Schopp L.H., Thomson D., Hathaway S.L., Mazurek M.O., Sanford-Martens T.C. (2008). Men with serious injuries: Relations among masculinity, age, and alcohol use. Rehabil. Psychol..

[16] Abbott D.H., Keverne E.B., Bercovitch F.B., Shively C.A., Mendoza S.P., Saltzman W., Snowdon C.T., Ziegler T.E., Banjevic M., Garland T. (2003). Are subordinates always stressed? A comparative analysis of rank differences in cortisol levels among primates. Horm. Behav..

[17] Piazza P.V., Le Moal M. (1998). The role of stress in drug self-administration. Trends Pharmacol. Sci..

[18] Brown S.A., Vik P.W., Patterson T.L., Grant I., Schuckit M.A. (1995). Stress, vulnerability and adult alcohol relapse. J. Stud. Alcohol.

[19] Wills T.A., Vaccaro D., McNamara G., Hirky A.E. (1996). Escalated substance use: A longitudinal grouping analysis from early to middle adolescence. J. Abnorm. Psychol..

[20] Enoch M.A. (2011). The role of early life stress as a predictor for alcohol and drug dependence. Psychopharmacology.

[21] Hernández J.L.S., Negrete D.B.D., Echeagaray F.W., Islas V.P. (2004). Factores psicosociales asociados con el abuso y la dependencia de drogas entre adolescentes: Análisis bivariados de un estudio de casos y controles. Salud Ment..

[22] Mazur A. (2005). Biosociology of Dominance and Deference.

[23] Wang F., Zhu J., Zhu H., Zhang Q., Lin Z., Hu H. (2011). Bidirectional Control of Social Hierarchy by Synaptic Efficacy in Medial Prefrontal Cortex. Science.

[24] Miczek K., Mutschler N., Mizcek K. (1996). Activational effects of social stress on IV cocaine self-administration in rats. Psychopharmacology.

[25] Toth I., Neumann I.D. (2013). Animal models of social avoidance and social fear. Cell Tissue Res..

[26] Coppens C.M., de Boer S.F., Steimer T., Koolhaas J.M. (2012). Impulsivity and aggressive behavior in Roman high and low avoidance rats: Baseline differences and adolescent social stress induced changes. Physiol. Behav..

[27] Coppens C.M., de Boer S.F., Buwalda B., Koolhaas J.M. (2014). Aggression and aspects of impulsivity in wild-type rats. Aggress. Behav..

[28] Boice R. (1971). Excessive water intake in captive Norway rats with scar-markings. Physiol. Behav..

[29] Yang C.R., Bai Y.Y., Ruan C.S., Zhou H.F., Liu D., Wang X.F., Shen L.J., Zheng H.Y. (2015). Enhanced Aggressive Behaviour in a Mouse Model of Depression. Neurotox. Res..

[30] Gross M., Pinhasov A. (2016). Chronic mild stress in submissive mice: Marked polydipsia and social avoidance without hedonic deficit in the sucrose preference test. Behav. Brain Res..

[31] Jupp B., Murray J.E., Jordan E.R., Xia J., Fluharty M., Shrestha S., Robbins T.W., Dalley J.W. (2016). Social dominance in rats: Effects on cocaine self-administration, novelty reactivity and dopamine receptor binding and content in the striatum. Psychopharmacology.

[32] Woodall K.L., Domeney A.M., Kelly M.E. (1996). Selective effects of 8-OH-DPAT on social competition in the rat. Pharmacol. Biochem. Behav..

[33] Lucion A., Vogel W.H. (1994). Effects of stress on defensive aggression and dominance in a water competition test. Integr. Physiol. Behav. Sci..

[34] Cruz F.C., Quadros I.M., Hogenelst K., Planeta C.S., Miczek K.A. (2011). Social defeat stress in rats: Escalation of cocaine and “speedball” binge self-administration, but not heroin. Psychopharmacology.

[35] Covington H.E., Miczek K.A. (2001). Repeated social-defeat stress, cocaine or morphine. Psychopharmacology.

[36] Quadros I.M.H., Miczek K.A. (2009). Two modes of intense cocaine bingeing: Increased persistence after social defeat stress and increased rate of intake due to extended access conditions in rats. Psychopharmacology.

[37] Yap J.J., Miczek K.A. (2007). Social defeat stress, sensitization, and intravenous cocaine self-administration in mice. Psychopharmacology.

[38] Marcolin M.L., Hodges T.E., Baumbach J.L., McCormick C.M. (2019). Adolescent social stress and social context influence the intake of ethanol and sucrose in male rats soon and long after the stress exposures. Dev. Psychobiol..

[39] Marcolin M.L., Baumbach J.L., Hodges T.E., McCormick C.M. (2020). The effects of social instability stress and subsequent ethanol consumption in adolescence on brain and behavioral development in male rats. Alcohol.

[40] Roeckner A.R., Bowling A., Butler T.R. (2017). Chronic social instability increases anxiety-like behavior and ethanol preference in male Long Evans rats. Physiol. Behav..

[41] van Erp A., Tachi N., Miczek K. (2001). Short or continuous social stress: Suppression of continuously available ethanol intake in subordinate rats. Behav. Pharmacol..

[42] Blanchard R.J., Hori K., Tom P., Blanchard D.C. (1987). Social structure and ethanol consumption in the laboratory rat. Pharmacol. Biochem. Behav..

[43] Pohorecky L.A. (2010). Acute novel stressors modify ethanol intake of psychosocially stressed rats. Pharmacol. Biochem. Behav..

[44] Ellison G., Levy A., Lorant N. (1983). Alcohol-preferring rats in colonies show withdrawal, inactivity, and lowered dominance. Pharmacol. Biochem. Behav..

[45] Filatova E.V., Egorov A.Y., Kutcher E.O., Shnitko T.A., Afanas’ev S.V. (2010). The influence of social conditions on the development of ethanol preference in rats. Dokl. Biol. Sci..

[46] Bahi A. (2013). Increased anxiety, voluntary alcohol consumption and ethanol-induced place preference in mice following psychosocial stress. Stress.

[47] Hilakivi-Clarke L., Lister R. (1992). Social-status and voluntary alcohol-consumption in mice-interaction with stress. Psychopharmacology.

[48] Kudryavtseva N., Madorskaya I., Bakshtanovskaya I. (1991). Social success and voluntary ethanol-consumption in mice of C57BL/6J and CBA/LAC strains. Physiol. Behav..

[49] Wolffgramm J., Heyne A. (1991). Social-behavior, dominance, and social deprivation of rats determine drug choice. Pharmacol. Biochem. Behav..

[50] Kudryavtseva N., Gerrits M.A.F.M., Avgustinovich D.F., Tenditnik M.V., Van Ree J.M. (2006). Anxiety and ethanol consumption in victorious and defeated mice; effect of kappa-opioid receptor activation. Eur. Neuropsychopharmacol..

[51] Keeney A., Hogg S. (1999). Behavioural consequences of repeated social defeat in the mouse: Preliminary evaluation of a potential animal model of depression. Behav. Pharmacol..

[52] Duncan E.A., Tamashiro K.L., Nguyen M.M., Gardner S.R., Woods S.C., Sakai R.R. (2006). The impact of moderate daily alcohol consumption on aggression and the formation of dominance hierarchies in rats. Psychopharmacology.

[53] Pohorecky L.A., Sweeny A. (2012). Amphetamine modifies ethanol intake of psychosocially stressed male rats. Pharmacol. Biochem. Behav..

[54] Heyne A. (1996). The development of opiate addiction in the rat. Pharmacol. Biochem. Behav..

[55] Mongillo D.L., Kosyachkova E.A., Nguyen T.M., Holmes M.M. (2014). Differential effects of chronic fluoxetine on the behavior of dominant and subordinate naked mole-rats. Behav. Brain Res..

[56] Endo T., Kakeyama M., Uemura Y., Haijima A., Okuno H., Bito H., Tohyama C. (2012). Executive Function Deficits and Social-Behavioral Abnormality in Mice Exposed to a Low Dose of Dioxin in Utero and via Lactation. PLoS ONE.

[57] Burke A.R., Watt M.J., Forster G.L. (2011). Adolescent social defeat increases adult amphetamine conditioned place preference and alters D2 dopamine receptor expression. Neuroscience.

[58] Yamaguchi Y., Lee Y.A., Kato A., Goto Y. (2017). The Roles of Dopamine D1 Receptor on the Social Hierarchy of Rodents and Nonhuman Primates. Int. J. Neuropsychopharmacol..

[59] Dalley J.W., Fryer T.D., Brichard L., Robinson E.S., Theobald D.E., Lääne K., Peña Y., Murphy E.R., Shah Y., Probst K. (2007). Nucleus Accumbens D2/3 Receptors Predict Trait Impulsivity and Cocaine Reinforcement. Science.

[60] Ersche K.D., Turton A.J., Chamberlain S.R., Müller U., Bullmore E.T., Robbins T.W. (2012). Cognitive dysfunction and Anxious-impulsive Personality Traits are Endophenotypes for Drug Dependence. Am. J. Psychiatry.

[61] Kaiser A., Bonsu J.A., Charnigo R.J., Milich R., Lynam D.R. (2016). Impulsive Personality and Alcohol Use: Bidirectional Relations Over One Year. J. Stud. Alcohol Drugs.

[62] Wongwitdecha N., Marsden C.A. (1996). Social isolation increases aggressive behaviour and alters the effects of diazepam in the rat social interaction test. Behav. Brain Res..

[63] Perry J.L., Carroll M.E. (2008). The role of impulsive behavior in drug abuse. Psychopharmacology.

[64] Cervantes M.C., Delville Y. (2007). Individual differences in offensive aggression in golden hamsters: A model of reactive and impulsive aggression?. Neuroscience.

[65] Coppens C.M., Coolen A., de Boer S.F., Koolhaas J.M. (2014). Adolescent social defeat disturbs adult aggression-related impulsivity in wild-type rats. Behav. Process..

[66] Vekovischeva O.Y., Semenova S.G., Verbitskaya E.V., Zvartau E.E. (2004). Effects of morphine and cocaine in mice with stable high aggressive and nonaggressive behavioral strategy. Pharmacol. Biochem. Behav..

[67] Morgan D., Grant K.A., Gage H.D., Mach R.H., Kaplan J.R., Prioleau O., Nader S.H., Buchheimer N., Ehrenkaufer R.L., Nader M.A. (2002). Social dominance in monkeys: Dopamine D2 receptors and cocaine self-administration. Nat. Neurosci..

[68] Nader M.A., Nader S.H., Czoty P.W., Riddick N.V., Gage H.D., Gould R.W., Blaylock B.L., Kaplan J.R., Garg P.K., Davies H.M. (2012). Social dominance in female monkeys: Dopamine receptor function and cocaine reinforcement. Biol. Psychiatry.

[69] Tarter R.E., Kirisci L., Kirillova G.P., Gavaler J., Giancola P., Vanyukov M.M. (2007). Social dominance mediates the association of testosterone and neurobehavioral disinhibition with risk for substance use disorder. Psychol. Addict. Behav..

[70] De Jong J.G., Wasilewski M., van der Vegt B.J., Buwalda B., Koolhaas J.M. (2005). A single social defeat induces short-lasting behavioral sensitization to amphetamine. Physiol. Behav..

[71] Carter J.S., Kearns A.M., Vollmer K.M., Garcia-Keller C., Weber R.A., Baker N.L., Kalivas P.W., Reichel C.M. (2020). Long-term impact of acute restraint stress on heroin self-administration, reinstatement, and stress reactivity. Psychopharmacology.

[72] Montagud-Romero S., Montesinos J., Pascual M., Aguilar M.A., Roger-Sanchez C., Guerri C., Miñarro J., Rodríguez-Arias M. (2016). Up-regulation of histone acetylation induced by social defeat mediates the conditioned rewarding effects of cocaine. Prog. Neuropsychopharmacol. Biol. Psychiatry.

[73] Rodríguez-Arias M., Montagud-Romero S., Rubio-Araiz A., Aguilar M.A., Martín-García E., Cabrera R., Maldonado R., Porcu F., Colado M.I., Miñarro J. (2017). Effects of repeated social defeat on adolescent mice on cocaine-induced CPP and self-administration in adulthood: Integrity of the blood-brain barrier. Addict. Biol..

[74] Goto T., Kubota Y., Tanaka Y., Iio W., Moriya N., Toyoda A. (2014). Subchronic and mild social defeat stress accelerates food intake and body weight gain with polydipsia-like features in mice. Behav. Brain Res..

[75] Goto T., Toyoda A. (2015). A Mouse Model of Subchronic and Mild Social Defeat Stress for Understanding Stress-induced Behavioral and Physiological Deficits. J. Vis. Exp..

[76] Calcaterra S.L., Beaty B., Mueller S.R., Min S.J., Binswanger I.A. (2014). The association between social stressors and drug use/hazardous drinking among former prison inmates. J. Subst. Abus. Treat..

[77] Maté G. (2012). Addiction: Childhood Trauma, Stress and the Biology of Addiction. J. Restor. Med..

[78] Alvarez F.J., Fierro I., del Río M.C. (2006). Alcohol-Related Social Consequences in Castille and Leon, Spain. Alcohol. Clin. Exp. Res..

[79] Perreault H.A., Semsar K., Godwin J. (2003). Fluoxetine treatment decreases territorial aggression in a coral reef fish. Physiol. Behav..

[80] Barbosa H.P., Lima-Maximino M.G., Maximino C. (2019). Acute fluoxetine differently affects aggressive display in zebrafish phenotypes. Aggress. Behav..

[81] Kohlert J.G., Mangan B.P., Kodra C., Drako L., Long E., Simpson H. (2012). Decreased aggressive and locomotor behaviors in Betta splendens after exposure to fluoxetine. Psychol. Rep..

[82] Hamilton T.J., Kwan G.T., Gallup J., Tresguerres M. (2016). Acute fluoxetine exposure alters crab anxiety-like behaviour, but not aggressiveness. Sci. Rep..

[83] Wolkers C., Serra M., Júnior A.B., Urbinati E.C. (2017). Acute fluoxetine treatment increases aggressiveness in juvenile matrinxã (Brycon amazonicus). Fish Physiol. Biochem..

[84] Ricci L.A., Melloni R.H. (2012). Repeated fluoxetine administration during adolescence stimulates aggressive behavior and alters serotonin and vasopressin neural development in hamsters. Behav. Neurosci..

[85] Coskun M., Zoroglu S. (2009). Efficacy and Safety of Fluoxetine in Preschool Children with Obsessive-Compulsive Disorder. J. Child Adolesc. Psychopharmacol..

[86] Liebowitz M.R., Turner S.M., Piacentini J., Beidel D.C., Clarvit S.R., Davies S.O., Graae F., Jaffer M., Lin S.-H., Sallee F.R. (2002). Fluoxetine in Children and Adolescents with OCD: A Placebo-Controlled Trial. J. Am. Acad. Child Adolesc. Psychiatry.

[87] Nielen M.M., Boer J.A.D. (2003). Neuropsychological performance of OCD patients before and after treatment with fluoxetine: Evidence for persistent cognitive deficits. Psychol. Med..

[88] Ruiz P., Calliari A., Pautassi R.M. (2018). Reserpine-induced depression is associated in female, but not in male, adolescent rats with heightened, fluoxetine-sensitive, ethanol consumption. Behav. Brain Res..

[89] McBride W.J., Murphy J.M., Lumeng L., Li T.K. (1988). Effects of Ro 15-4513, fluoxetine and desipramine on the intake of ethanol, water and food by the alcohol-preferring (P) and -nonpreferring (NP) lines of rats. Pharmacol. Biochem. Behav..

[90] Naranjo C.A., Poulos C.X., Bremner K.E., Lanctot K.L. (1994). Fluoxetine attenuates alcohol intake and desire to drink. Int. Clin. Psychopharmacol..

[91] Kakeyama M., Endo T., Zhang Y., Miyazaki W., Tohyama C. (2014). Disruption of paired-associate learning in rat offspring perinatally exposed to dioxins. Arch. Toxicol..

[92] Markowski V.P., Cox C., Preston R., Weiss B. (2002). Impaired cued delayed alternation behavior in adult rat offspring following exposure to 2,3,7,8-tetrachlorodibenzo-p-dioxin on gestation day 15. Neurotoxicol. Teratol..

[93] He X.X., Nebert D.W., Vasiliou V., Zhu H., Shertzer H.G. (1997). Genetic differences in alcohol drinking preference between inbred strains of mice. Pharmacogenetics.

[94] Dickerson A.S., Ransome Y., Karlsson O. (2019). Human prenatal exposure to polychlorinated biphenyls (PCBs) and risk behaviors in adolescence. Environ. Int..

[95] Jankowska E., Bidzinski A., Kostowski W. (1994). Alcohol drinking in rats treated with 5,7-dihydroxytryptamine: Effect of 8-OH-DPAT and tropisetron (ICS 205-930). Alcohol.

[96] McKenzie-Quirk S.D., Miczek K.A. (2003). 5-HT1A agonists: Alcohol drinking in rats and squirrel monkeys. Psychopharmacology.

[97] West C.H., Boss-Williams K.A., Weiss J.M. (2011). Effects of fenfluramine, 8-OH-DPAT, and tryptophan-enriched diet on the high-ethanol intake by rats bred for susceptibility to stress. Alcohol.

[98] Berman M., Taylor S., Marged B. (1993). Morphine and human aggression. Addict. Behav..

[99] Spiga R., Cherek D.R., Roache J.D., Cowan K.A. (1990). The Effects of Codeine on Human Aggressive Responding. Int. Clin. Psychopharmacol..

[100] Gardner E.L. (2011). Addiction and Brain Reward and Antireward Pathways. Adv. Psychosom. Med..

[101] Baik J.H. (2013). Dopamine signaling in reward-related behaviors. Front. Neural Circuits.

[102] Hasbi A., Perreault M.L., Shen M., Fan T., Nguyen T., Alijaniaram M., Banasikowski T.J., Grace A.A., O’Dowd B.F., Fletcher P.J. (2018). Activation of Dopamine D1-D2 Receptor Complex Attenuates Cocaine Reward and Reinstatement of Cocaine-Seeking through Inhibition of DARPP-32, ERK, and ΔFosB. Front. Pharmacol..

[103] Edwards S., Whisler K.N., Fuller D.C., Orsulak P.J., Self D.W. (2007). Addiction-related alterations in D1 and D2 dopamine receptor behavioral responses following chronic cocaine self-administration. Neuropsychopharmacology.

[104] Dyr W., McBride W.J., Lumeng L., Li T.K., Murphy J.M. (1993). Effects of D1 and D2 dopamine receptor agents on ethanol consumption in the high-alcohol-drinking (HAD) line of rats. Alcohol.

[105] Kreisler A.D., Terranova M.J., Somkuwar S.S., Purohit D.C., Wang S., Head B.P., Mandyam C.D. (2020). In vivo reduction of striatal D1R by RNA interference alters expression of D1R signaling -related proteins and enhances methamphetamine addiction in male rats. Brain Struct. Funct..

[106] Ersche K.D., Roiser J.P., Abbott S., Craig K.J., Müller U., Suckling J., Ooi C., Shabbir S.S., Clark L., Sahakian B.J. (2011). Response Perseveration in Stimulant Dependence Is Associated with Striatal Dysfunction and Can Be Ameliorated by a D2/3 Receptor Agonist. Biol. Psychiatry.

[107] Kanen J.W., Ersche K.D., Fineberg N.A., Robbins T.W., Cardinal R.N. (2019). Computational modelling reveals contrasting effects on reinforcement learning and cognitive flexibility in stimulant use disorder and obsessive-compulsive disorder: Remediating effects of dopaminergic D2/3 receptor agents. Psychopharmacology.

[108] O’Malley S.S., Jaffe A.J., Chang G., Schottenfeld R.S., Meyer R.E., Rounsaville B. (1992). Naltrexone and Coping Skills Therapy for Alcohol Dependence. Arch. Gen. Psychiatry.

[109] Volpicelli J.R., Alterman A.I., Hayashida M., O’Brien C.P. (1992). Naltrexone in the Treatment of Alcohol Dependence. Arch. Gen. Psychiatry.

[110] Insel T.R., Pickar D. (1983). Naloxone administration in obsessive-compulsive disorder: Report of two cases. Am. J. Psychiatry.

